# Identification of Sliding Hiatus Hernia by High-Resolution Manometry and Upper Gastrointestinal Endoscopy in Patients with Gastro-Oesophageal Reflux Disease

**DOI:** 10.3390/jcm11236906

**Published:** 2022-11-23

**Authors:** Antoni Stadnicki, Józef Kurek, Ewa Klimacka-Nawrot, Anna Stadnicka, Katarzyna Rerych

**Affiliations:** 1 Department of Basic Biomedical Science, Faculty of Pharmaceutical Sciences in Sosnowiec, Medical University of Silesia, 41-200 Sosnowiec, Poland; 2 Section of Gastrointestinal Motility, Multidisciplinary Hospital, 43-600 Jaworzno, Poland; 3 Department of General, Endocrinology and Oncology Surgery, Multidisciplinary Hospital, 43-600 Jaworzno, Poland; 4 Unit of Health, University of Economics and Innovation, 20-209 Lublin, Poland

**Keywords:** upper GI endoscopy, GORD, hiatus hernia, HRM, Nissen fundoplication, sensitivity, CT-scan

## Abstract

Background/Aim: The aim of this study was to compare high-resolution manometry (HRM) and upper gastrointestinal (GI) endoscopy as diagnostic utilities in detecting a sliding hiatus hernia in patients with gastro-oesophageal reflux disease (GORD) symptoms. Material and Methods: For both diagnostic modalities, the data obtained from 31 patients (20 females; mean age 48.2) who qualified for Nissen fundoplication were analysed using oesophageal pressure topography in line with the Chicago Classification. Confirmation of hiatus hernia during the surgery was considered the gold standard. HRM protocol involved 10 consecutive boluses of 10 mL of water. Results: Sliding hiatus hernia was confirmed intraoperatively in 29 out of 31 patients. In 14 patients, hiatus hernia was detected in HRM, while 19 patients were found to have hiatus hernia by upper GI endoscopy before surgery. No false positive results were obtained in HRM, while 15 false negative results were shown. In upper GI endoscopy, false positive data were observed in 1 patient, while false negative results were found in 10 patients. Thus, the sensitivity of HRM in detecting hiatus hernia was 48% (95%CIs: 29–67%), and sensitivity of upper GI endoscopy was 66% (95%CIs: 46–82%). It was not possible to assess the specificity of HRM or upper GI endoscopy because only 2 of 31 patients had no hiatus hernia during fundoplication (gold standard). False negative results (sensitivity) were not significantly different between compared diagnostic modalities HRM and upper GI endoscopy (52% vs. 34%, respectively, *p* = 0.29). Conclusions: Due to poor sensitivity, both modalities, i.e., HRM and upper GI endoscopy, are not reliable tools to diagnose sliding hiatus hernia in patients with GORD symptoms.

## 1. Introduction

There are three different types of hiatus hernia. A sliding hiatus hernia (type I) is the most common type and accounts for 95% of all hiatus hernias. A sliding hernia occurs when part of the stomach with the gastro-oesophageal junction slides up through the hiatus opening in the diaphragm. In para-oesophageal hernia, displacement of the stomach fundus to the chest takes place with a properly fixed gastro-oesophageal junction. The third type of hiatus hernia is known as mixed hernia [1]. Usually, in the case of the most common asymptomatic hiatus hernias, no treatment is required. Nonetheless, a sliding hiatus hernia is a significantly contributing factor predisposing to the development of gastro-oesophageal reflux disease (GORD) [2]. Typical GORD symptoms include heartburn or/and regurgitations which occur as result of incompetent function of gastro-oesophageal barrier.

GORD appears to be one of the most common diseases in Europe. The following factors play a crucial role in the pathogenesis of GORD: an ineffective gastro-oesophageal barrier, which consists of decreased lower oesophageal sphincter (LOS) tone and/or co-existence of hiatus hernia; an increased frequency of transient lower oesophageal sphincter relaxations (TLOSRs), and delayed gastric emptying as well as impaired oesophageal motility [3,4]. With regard to sliding hiatus hernia, the LOS remains in tonic dilatation, as a result of a change in anatomical conditions in the form of displacement of the lower oesophagus to the chest, where the pressure is negative (approximately −6 mm Hg) as compared with positive pressure inside the abdominal cavity. Negative pressure impedes LOS closure, which is critical for the constant oesophageal exposure to the acidic gastric content [5].

A hiatus hernia is typically identified during barium swallow examination, upper GI endoscopy, and high-resolution manometry. Current data related to accuracy of HRM and upper GI endoscopy in diagnosing hiatus hernia are equivocal. The purpose of the present study was to compare the value of HRM and upper GI endoscopy in detecting sliding hiatus hernia in patients with GORD symptoms qualified for laparoscopic Nissen fundoplication.

## 2. Materials and Methods

Thirty-one consecutive patients (twenty women, eleven men; mean age 48.2 ± 14.07) with long-term/persistent GORD were enrolled in this prospective study. All patients had complained of typical GORD symptoms such as heartburn and/or regurgitations which did not resolve despite treatment with a proton pump inhibitor (PPI). Prior to study enrolment, the duration of GORD symptoms experienced by patients ranged from 2 years to 10 years. In addition, 5 patients complained of non-cardiac chest pains (NCCP), and 4 patients complained of nausea/vomiting. The body mass index (BMI) was calculated for each patient as weight in kg/(height in m)^2^. The mean BMI before fundoplication was 25.36 ± 3.54 kg/m^2^. Thirteen patients had normal BMI (18.5–24.99 kg/m^2^), one patient was underweight (BMI < 18.49 kg/m^2^), whereas thirteen patients were overweight (BMI > 25–29.9 kg/m^2^) and four patients were obese (BMI ≥ 30 kg/m^2^).

All patients had undergone HRM and upper GI endoscopy at the Section of Gastrointestinal Motility in Multidisciplinary Hospital in Jaworzno and were subsequently qualified for laparoscopic Nissen fundoplication. All patients were informed to discontinue antireflux (e.g., H_2_-blockers and PPIs) and prokinetic medicines, calcium channel blockers, nitrates, and anticholinergic drugs a minimum of 72 h before manometric examination. Approval from the local Ethics Committee of the Medical University of Silesia (approval number: KNW/0022/KB1/105/I/10) was obtained on 12 October 2010. All study participants gave written informed consent. The study was conducted according to the Declaration of Helsinki.

Despite negative results for hiatus hernia in upper GI endoscopy and HRM, all patients qualified for Nissen fundoplication due to long-term GORD symptoms, which did not resolve despite a standard therapy with PPIs and, due to incompetent gastro-oesophageal barrier IE, decreased LOS pressure/hypotensive LOS. Low resting pressure in the LOS strongly favours the development of GORD. In our study, the detection of hiatus hernia upon upper GI endoscopy and/or HRM only strengthened recommendations for surgical treatment/ fundoplication. In fact, resistance to PPI therapy, especially in younger populations of patients with long-term perspectives of continuous therapy with PPIs, was one of the most important factors while considering patient’s eligibility for a surgical intervention.

### 2.1. High-Resolution Manometry and Study Protocol

High-resolution manometry (Sierra Scientific Instruments, Angeles, CA, USA) with a solid-state catheter (36 circumferentially incorporated sensors spaced at 1 cm intervals) were used. ManoView Z version 2.0.1. (Sierra Scientific Instruments, Los Angeles, CA, USA) with updated software for Chicago classification of oesophageal motility disorders v 3.0 and pressure topography was utilised to analyse the data obtained from individual patients.

Assessment of oesophagogastric junction (OGJ) relaxation pressure was expressed as integrated relaxation pressure (IRP) during swallow-induced 4 continuous or non-continuous seconds of lowest mean pressures. While efficacy of antireflux barrier was assessed by measuring resting OGJ pressures during period free from swallowing (minimal and mean basal OGJ pressure).

**Figure 1:**
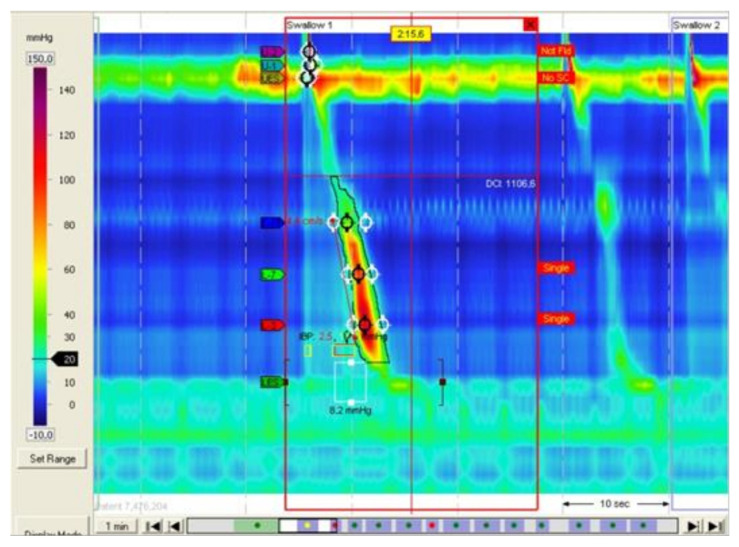
HRM recording in a patient with a hiatus hernia with a clear separation observed between LOS and the crura of the diaphragm.

Hiatus hernia was identified by analysis of gastro-oesophageal junction morphology using high-resolution manometry topographic pressure plots. The evidence of hiatus hernia in HRM was a separation between an area of high pressure corresponding to OGJ and that of the crura of the diaphragm (Figure 1).

The size of hiatus hernia was measured as a distance spanning between the centre of the LOS and the high-pressure zones at the level corresponding to the crura of the diaphragm.

Intraoperative diagnosis of hiatus hernia was considered the gold standard. The measurements of hiatus hernia were performed using the Covidien laparoscopic grasper with the width of 20 mm after opening. Both the length and width of hernia gate were measured after dissecting the bag and its contents and visualising the entire crura of diaphragm.

HRM was performed on an empty stomach in individual study participants who remained in a sitting position. Following a trans-nasal catheter insertion, a 5 min period of adaptation was required after which OGJ baseline pressure was captured during 30 s without swallowing (Landmark). Subsequently, 10 consecutive boluses of 10 mL of water administered by a syringe every 30 s and water swallows were registered/recorded to evaluate oesophageal peristalsis. Two experienced investigators (K.R. and A.S.) analysed the data. Both investigators/authors were blinded to the endoscopic results at the time of analysis. Notably, the modified Chicago classification protocol for HRM was used, which included a series of ten swallows of 10 mL of water (instead of 5 mL of water as recommended by Chicago classification) administered to patients in an upright/sitting position (instead of supine position).

The reference range for manometric parameters corresponding to oesophageal motility and OGJ pressures were established as a consensus between manufacturer’s recommendations and the Chicago classification criteria.

The sliding hiatus hernia was detected during upper GI endoscopy when the “hernial sac” was at least 2 cm from the Z line to the diaphragm impression. Upper GI endoscopies were performed using Olympus Evis, Extera II. Upper GI endoscopy was performed by A.S., who has good experience in endoscopic examinations.

### 2.2. Statistical Analysis

The sensitivity of HRM and upper GI endoscopy in detecting a hiatus hernia were evaluated and presented with the 95% confidence interval in all patients qualified for Nissen fundoplication. However, it was not possible to assess/calculate specificity because only 2 of the 31 patients had no hiatus hernia during operation. McNemar’s test was performed to evaluate probability of agreement between the positive results of two tests among same patients. Chi-squared tests with Yates correction were performed to compare the proportion of false negative results between the two tests. *p* values < 0.05 were considered statistically significant for all tests.

## 3. Results

In total, 29 patients out of 31 were found to have sliding hiatus hernia during surgery. Fourteen patients had manometric criteria for hiatus hernia; mean hiatus hernia size: 2.4 ± 0.57 cm. HRM pattern was characterized by an oesophagogastric junction (OGJ) with separation of the LOS and crura of the diaphragm, which was associated with a significantly lower OGJ pressure. Nineteen patients were found to have hiatus hernia by upper GI endoscopy before surgery (the data were shown in Table 1).

In HRM, there were 15 false negative results shown; the sensitivity of HRM in detecting hiatus hernia was 48% (95%CIs: 29–67%). In the upper GI endoscopy, false negative results were found in 10 patients indicating sensitivity of 66% (95%CIs: 46–82%) for upper GI endoscopy in detection of hiatus hernia. False negative results (sensitivity) were not significantly different between compared diagnostic modalities HMR and upper GI endoscopy (52% vs. 34%, respectively, *p* = 0.29).

## 4. Discussion

The prevalence of hiatus hernia in patients with GORD is very high [6]. In the present study, during laparoscopic surgery, hiatus hernia was found in slightly over 90% of patients. In addition, it should be noted that assessment of hiatus hernia size is critical for patients with GORD qualified for endoscopic treatment modalities as those with hiatus hernia greater than 2 cm are usually excluded [7,8].

Before laparoscopic surgery hiatus hernia is usually evaluated by upper GI endoscopy and high-resolution manometry. In fact, barium swallow can be omitted as a basic diagnostic test before laparoscopic antireflux and hiatus hernia surgery. In a previous study, Linke at al examined patients with GORD in whom barium swallow and upper GI endoscopy had been performed before laparoscopic surgery showing that a barium swallow did not provide any further essential/meaningful information [9]. The HRM examination enables precise determination of the size of hernias, including those of small sizes. The high-pressure zone (HPZ) recorded during the HRM study in healthy subjects covers the LOS and the diaphragm. However, the presence of the so-called dual high-pressure zone (DHPZ) indicates the presence of a hiatus hernia, where the LOS is the proximal zone, and the distal band of elevated pressure corresponds to the compression of the crura of the diaphragm on the displaced part of the stomach. The laparoscopic fundoplication procedure using the Nissen method is primarily aimed at eliminating the mechanical cause of the disease by regulating the LOS pressure and, in the event of a hiatus hernia, performing a corrective, plastic surgery [10,11].

The data on sensitivity and specificity of both upper GI endoscopy and HRM in detecting hiatus hernia are limited. Recently, Khajanchee et al. conducted a retrospective study to compare the specificity and sensitivity of HRM and upper GI endoscopy in the diagnosis of sliding hiatus hernia in patients with GORD [12]. In our study, as in the study by Khajanchee et al., a presence of a hernia during laparoscopic Nissen fundoplication was considered the gold standard. Khajanchee et al. demonstrated that the sensitivity and specificity of HRM for detection of sliding hernia are 52% and 95%, respectively. The results of our study showed that the sensitivity of the HRM in the diagnosis of hiatus hernia was 48%, which is in agreement with Khajanchee et al.’s results. Our study also demonstrated a slightly better sensitivity of the endoscopic examination as compared with the HRM (66%).

However, there are no unified/harmonised diagnostic criteria employed by clinicians and subjective variations in the assessment of hiatus hernia by upper GI endoscopy, which makes this method less useful for an objective/unbiased diagnosis [9]. In contrast, Weijenborg et al. found the high diagnostic accuracy of hiatus hernia detection with HRM, showing a sensitivity of HRM as 92% and a specificity of 95%, which exceeded the sensitivity of upper GI endoscopy or radiography alone (both 73%) [13]. However, these authors retrospectively compared patients’ HRM records, upper GI endoscopy reports, and barium esophagograms data without surgical verification.

Although medical history and lack of response to PPI therapy are insufficient to make a conclusive diagnosis of GORD, they are of value in determining need for further investigation. Upper GI endoscopy provides supportive evidence of erosive oesophagitis (as Los Angeles grades A–D), segment of Barrett’s oesophagus, and it is necessary to exclude diseases like cancer or peptic strictures, although normal endoscopy does not exclude GORD.

Twenty-four-hour pH meter examination was regarded as the gold standard to diagnose GORD; however, the development of multichannel intraluminal pH-impedance has improved the accuracy to detect and characterise the type of reflux. Multi-channel intraluminal impedance pH (MII-pH) seems to be the most sensitive tool to evaluate patients with both typical and atypical reflux symptoms [14]. Alternatively, wireless oesophageal pH monitoring (the wireless pH Bravo capsule or its modification) has similar value for testing oesophageal acid exposure as compared with conventional catheter monitoring system. The Bravo capsule is a feasible, safe, and well-tolerated device [15,16].

The results of our study have shown no significant discordance between HRM and upper GI endoscopy, which is in agreement with the results obtained by Khajanchee et al. Thus, one method cannot be used to confirm a diagnosis of hiatus hernia in patients who are negative for hiatus hernia by the other method. Khajanchee et al. suggested that the more frequent diagnosis of hernia during antireflux surgery as compared with the HRM examination is due to gas (CO_2_) insufflation of the abdominal cavity, hence the visualisation/enlargement of small hernias [12]. However, endoscopic evaluations of hernia may also be influenced by excessive insufflation of the stomach [17]. As demonstrated by Khajanchee et al. and the results of our study, both diagnostic modalities, i.e., HRM and upper GI endoscopy failed to detect hiatus hernia in a large cohort /proportion of patients with GORD.

Reliable pre-operative detection of hiatus hernia along with its type can be achieved by computed tomography (CT) imaging in patients with GORD. As early as in 1981, Lindell and Bernardino (TX, USA) found that CT scan (with a contrast solution, 5% Gastrografin) performed in a patient in a supine position may reveal hiatus hernia, phrenic ampulla, and oesophageal reflux [18]. Later studies revealed that preoperative hiatus hernia may be easily evaluated by CT imaging [19,20]. However, intra-operative measurement of the area of the hiatus surface by this technique is much more difficult [19]. Recently, in their large retrospective study, Karatay et al. estimated hiatus surface area and other measurements (hiatus anterior–posterior (A–P) diameter and hiatus transverse diameter) in patients with GORD using thorax and abdominal CT images [21]. They demonstrated that a CT scan is a valuable tool for preoperative detection and measurements of hiatus hernia in patients with GORD. In fact, CT scans should routinely be performed in patients with GORD before operation.

The limitations of our study include a small study sample—the total number of enrolled patients was 31. A small number of recruited patients significantly reduces the real clinical value of analysed data and limits the interpretation of study results obtained during HRM and upper GI endoscopy as diagnostic methods for the detection of hiatus hernia. Other study limitations include the following: lack of CT imagining as additional diagnostic modality for a detection of hiatus hernia and lack of preoperative pH oesophageal monitoring or wireless capsule reflux testing to confirm pathological oesophageal acid exposure.

Moreover, it should be noted that the authors of the current study utilised the modified Chicago protocol which included swallowing boluses of 10 mL of water (versus 5 mL per established Chicago protocol) in a sitting position (versus supine position). Although the supine position eliminates the impact of gravity on swallowing and it is considered the gold Chicago standard, demonstrating the actual performance of oesophageal motility, the upright position has the advantage of reflecting a real-life scenario, i.e., a patient consuming food and liquids in a natural position. The methodology utilised in the current study represents a deviation from the well-established Chicago protocol and it is considered one of study limitations. Nonetheless, Pandolfino et al., did not demonstrate statistically significant differences in nadir EGI relaxation among three volume swallows such as 5 mL, 10 mL and 20 mL swallows [22].

## 5. Conclusions

Data from various studies indicate there is a correlation between hiatal hernia and GORD. Thus, the accurate diagnosis of sliding hiatal hernia is a significant indication for an antireflux surgery. Our prospective study is one of the first to provide an evaluation of hiatal hernia and EGJ parameters by HRM in patients with GORD symptoms before fundoplication. However, due to poor sensitivity, HRM is not a reliable tool to diagnose sliding hiatus hernia in patients with GORD symptoms. In other studies, a significant number of false negative results and relatively poor likelihood ratios for a negative test limit the HRM for ruling in sliding hiatal hernias. Thus, randomised, prospective multicentre studies are needed to fully evaluate HRM, upper GI endoscopy, and CT imaging as diagnostic modalities and to conclude which method is diagnostically the most valuable for the detection of hiatal hernia in patients with GORD symptoms before antireflux surgery. In addition, pH monitoring should be used to detect GORD and to enable the most effective management of GORD.

## Figures and Tables

**Table 1:** Detection of hiatus hernia by either HRM or upper GI endoscopy, or both.

Diagnosis of Hiatus Hernia	HRM	Upper GI Endoscopy	Gold Standard
True positive	14	19	29
True negative	2	1	2
False positive	0	1	0
False negative	15	10	0

## Data Availability

Medline, Pubmed and Embase were searched for relevant studies.
